# Quality and acceptability of measures of exercise adherence in musculoskeletal settings: a systematic review

**DOI:** 10.1093/rheumatology/kew422

**Published:** 2016-12-23

**Authors:** Sionnadh McLean, Melanie A. Holden, Tanzila Potia, Melanie Gee, Ross Mallett, Sadiq Bhanbhro, Helen Parsons, Kirstie Haywood

**Affiliations:** 1 Faculty of Health and Wellbeing, Collegiate Campus, Sheffield Hallam University, Sheffield; 2 Arthritis Research UK Primary Care Centre, Keele University, Keele; 3 Centre for Health and Social Care Research, Collegiate Campus, Sheffield Hallam University, Sheffield; 4 Clinical Trials Unit; 5 Royal College of Nursing Research Institute, Warwick Medical School, Warwick University, Coventry, UK

**Keywords:** acceptability, adherence, exercise, measurement, musculoskeletal, physical activity, quality, systematic review

## Abstract

**Objective.** To recommend robust and relevant measures of exercise adherence for application in the musculoskeletal field.

**Method.** A systematic review of measures was conducted in two phases. Phase 1 sought to identify all reproducible measures used to assess exercise adherence in a musculoskeletal setting. Phase 2 identified published evidence of measurement and practical properties of identified measures. Eight databases were searched (from inception to February 2016). Study quality was assessed against the Consensus-based Standards for the Selection of Health Measurement Instruments guidelines. Measurement quality was assessed against accepted standards.

**Results.** Phase 1: from 8511 records, 326 full-text articles were reviewed; 45 reproducible measures were identified. Phase 2: from 2977 records, 110 full-text articles were assessed for eligibility; 10 articles provided evidence of measurement/practical properties for just seven measures. Six were exercise adherence-specific measures; one was specific to physical activity but applied as a measure of exercise adherence. Evidence of essential measurement and practical properties was mostly limited or not available. Assessment of relevance and comprehensiveness was largely absent and there was no evidence of patient involvement during the development or evaluation of any measure.

**Conclusion.** The significant methodological and quality issues encountered prevent the clear recommendation of any measure; future applications should be undertaken cautiously until greater clarity of the conceptual underpinning of each measure is provided and acceptable evidence of essential measurement properties is established. Future research should seek to engage collaboratively with relevant stakeholders to ensure that exercise adherence assessment is high quality, relevant and acceptable.

Rheumatology key messages

- Current measures of exercise adherence for musculoskeletal populations are of poor quality.
- New measures of exercise adherence for musculoskeletal populations require a collaborative approach.

## Introduction

Musculoskeletal (MSK) disorders are burdensome [1]. For many, the associated progressive functional limitation in everyday activities, including paid employment, results in significant financial costs for individuals and society [2, 3]. Increasing age and lifestyle factors such as obesity and physical inactivity negatively impact MSK disorders [4, 5]; the ageing population and increasingly sedentary lifestyles suggest that the disease burden will continue to increase [4].

Exercise and physical activity (EPA) can reduce pain, improve physical dysfunction and enhance quality of life for individuals with MSK disorders [6–10]; clinical guidelines advocate EPA within long-term management strategies [3, 11–13]. Physical activity is defined as any bodily movement produced by skeletal muscle that results in energy expenditure and includes occupational, sporting and household activities [14]. Exercise, a subset of physical activity, is specific, structured, planned and repetitive [14]. In this article exercise indicates therapeutic EPA aimed at reducing MSK symptoms.

An individual’s ability to adhere to recommended exercise, defined as the extent to which a person’s behaviour corresponds with agreed recommendations from a healthcare provider, is important for success [15, 16]. Patients who adhere to regular exercise are less likely to progress to recurrent, persistent or disabling problems [17, 18]. Increasing adherence may give greater patient benefit than improving aspects of the intervention itself [16]. Adherence to prescribed exercise is frequently reported as < 50% [19–22]. Non-adherence may negatively impact treatment effectiveness and efficiency, therapeutic relationships, waiting times and cost of care [23–25]. Numerous strategies for increasing exercise adherence have been identified but their effectiveness is uncertain and guidance for best practice does not exist [26, 27]. Consequently, development and evaluation of exercise adherence interventions is essential [28]; however, guidance for the assessment of exercise adherence in MSK clinical trials or routine practice settings does not exist.

There is wide variation in the assessment of exercise adherence [29, personal communication, R. Frost, Glasgow Caledonian University]. Where large numbers of assessment approaches exist, structured reviews of the quality and acceptability of different approaches are essential to informing selection [30, 31]. This review seeks to identify all clearly reported and reproducible measures of exercise adherence applied in published studies of patients with MSK disorders, and to evaluate these measures against a transparent appraisal framework.

## Methods

This two-phase systematic review was reported in accordance with the Preferred Reporting Items for Systematic Reviews and Meta-Analyses guidelines [32]. Phase 1 identified clearly reported and reproducible measures of exercise adherence in published MSK studies. Phase 2 reviewed published and unpublished evidence of measurement and practical properties for shortlisted measures. Study and measurement quality were assessed against the Consensus-based Standards for the Selection of health Measurement Instruments (COSMIN) checklist [31, 33, 34], and a transparent appraisal framework [35], respectively.

### Phase 1: identifying measures of exercise adherence

A search strategy was developed to identify methods used to assess exercise adherence in MSK settings (see search strategy for phase 1 in Supplementary Data, available at *Rheumatology* Online, and study protocol [36]); all study types were included. Eight databases were searched (from inception to February 2016): Medline, SPORTDiscus, CINAHL Plus, PsycINFO, AMED, Cochrane Library, Embase and the Web of Science.

Titles, abstracts and full text articles were independently screened for inclusion by two reviewers from five (S.Mc., M.H., R.M., T.P., S.B.). Disagreement was discussed with a third independent reviewer from six (S.Mc., M.H., R.M., T.P., S.B., K.H.).

Articles were included if they focused on adults with an MSK disorder receiving therapeutic exercise in any setting, and for which assessments of adherence to exercise [patient- or clinician-reported or exercise diaries (if converted to an adherence scale)] were completed. Studies were excluded if they were not written in English or if participants were healthy volunteers, <18 years old, or with non-MSK conditions.

Reproducible measures of exercise adherence (i.e. the original measure could be located, had an appropriate citation or was reproducible based on information supplied by the author) [37, 38] were listed and categorized as clinician- or patient-reported. Performance measures (i.e. muscle strength, joint range of movement), performance of exercise technique and session attendance were excluded as proxy measures of adherence. Accelerometers and pedometers were excluded because they are primarily performance measures and measure motion rather than adherence.

### Phase 2: evidence of quality and acceptability

Separate searches were conducted in the above databases for each shortlisted measure. Where the result set for a measure exceeded 50, a sensitive search filter for the identification of studies reporting evidence of measurement and/or practical properties was additionally applied [39] (Search strategy for phase 2 in Supplementary Data, available at *Rheumatology* Online). The developers of specific measures were also contacted to request additional evidence of measurement evaluation. Titles, abstracts and full text articles were independently assessed by two reviewers from four (M.H., T.P., R.M., S.Mc.); a third reviewer resolved any disagreements (K.H.). Reference lists of included articles were reviewed for additional published articles. English language articles were included if they provided evidence of assessment development and/or evaluation of the named measure(s) in an MSK population.

#### Data extraction and inter-rater reliability

A data extraction form informed by earlier reviews [35] and the COSMIN checklist [31, 34] was used to capture study-specific (population, intervention and setting) and measurement-specific information: reliability (internal consistency, test–retest, intra-/inter-tester, measurement error); validity [content, structural validity (dimensionality), construct (evidence of explicit hypothesis testing); criterion]; responsiveness (criterion-/construct-based); interpretability (e.g. evidence of minimal important change); data precision (data quality, end effects); and evidence of where Item Response Theory models were applied. Extraction for practical properties included acceptability (relevance and respondent burden) and feasibility (clinician burden, including cost, time to complete and score) [30, 31, 34]. The extent of patient involvement in measurement development and/or application was also sought [35].

In accordance with the COSMIN checklist, study methodological quality was evaluated per measurement property and rated on a four-point scale (excellent, good, fair, poor); quality was determined by the lowest checklist rating per measurement property [31, 34]. Following a group training session, four primary reviewers (S.Mc., M.H., T.P., R.M.) independently undertook data extraction and applied the checklist. The reviewers were clinicians and/or researchers with little experience in assessing measurement properties and no previous exposure to the COSMIN checklist. The inter-rater agreement (percentage agreement) between two reviewers was evaluated for all included articles. Where disagreement existed, consensus was sought through a third, experienced reviewer (K.H.) who independently reviewed all articles.

#### Data synthesis

Data were qualitatively synthesized to determine the overall quality and acceptability of each measure [30, 33]. Synthesis considered the following: study methodological quality (COSMIN scores); number of studies reporting specific evidence per measure; results for each measurement and practical property per measure; and consistency between studies [33]. The overall quality of a measurement property was reported as: adequate (+), not adequate (−), conflicting (±), or unclear (?). Levels of evidence for the overall quality of each measurement property were further defined to indicate strong, moderate, limited, conflicting or unknown evidence [33].

## Results

### Identification of studies and measures

#### Phase 1

**Fig. 1:**
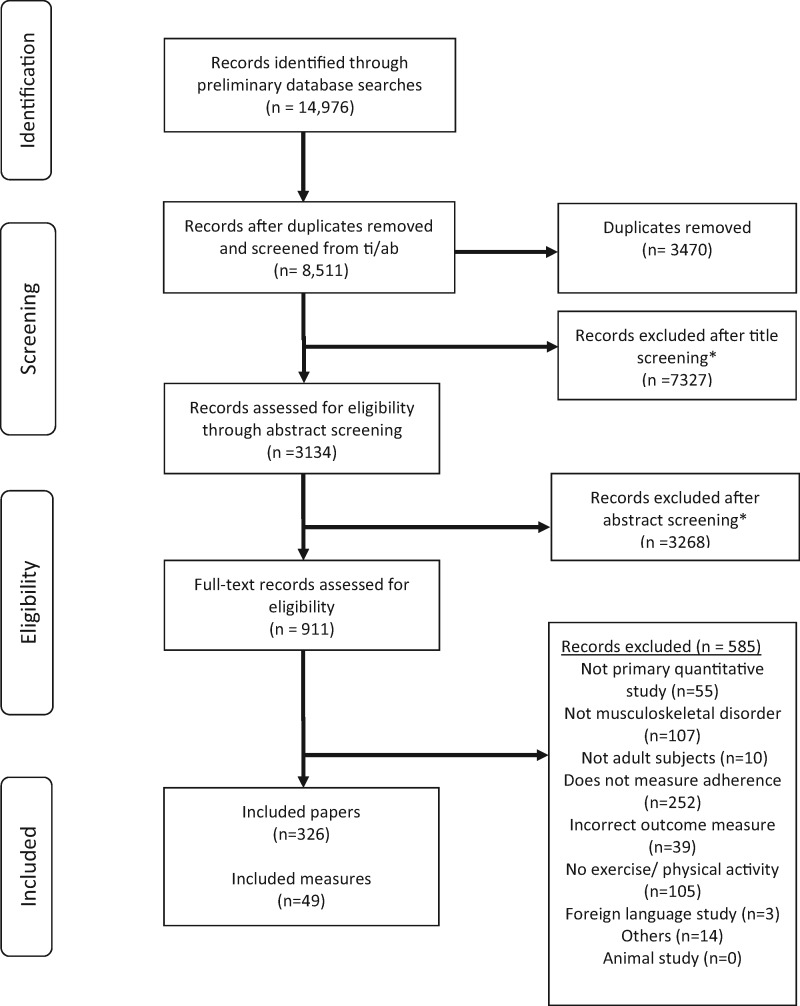
PRISMA flowchart for phase 1 of the systematic review

Following removal of duplicates, 8511 records were identified. Following title and abstract screening 326 full-text articles were retrieved and reviewed in full (Fig. 1).

A total of 234 approaches to measuring exercise adherence were identified. These included the following: exercise logs and diaries (n = 107); unnamed questionnaires or scales (n = 53); clearly described or named questionnaires or scales (n = 49); interviews (n = 17); and calendars or postcards (n = 8). Only the 49 clearly described and reproducible or named questionnaires or scales were included (Supplementary Table S1, available at *Rheumatology* Online).

#### Phase 2

**Fig. 2:**
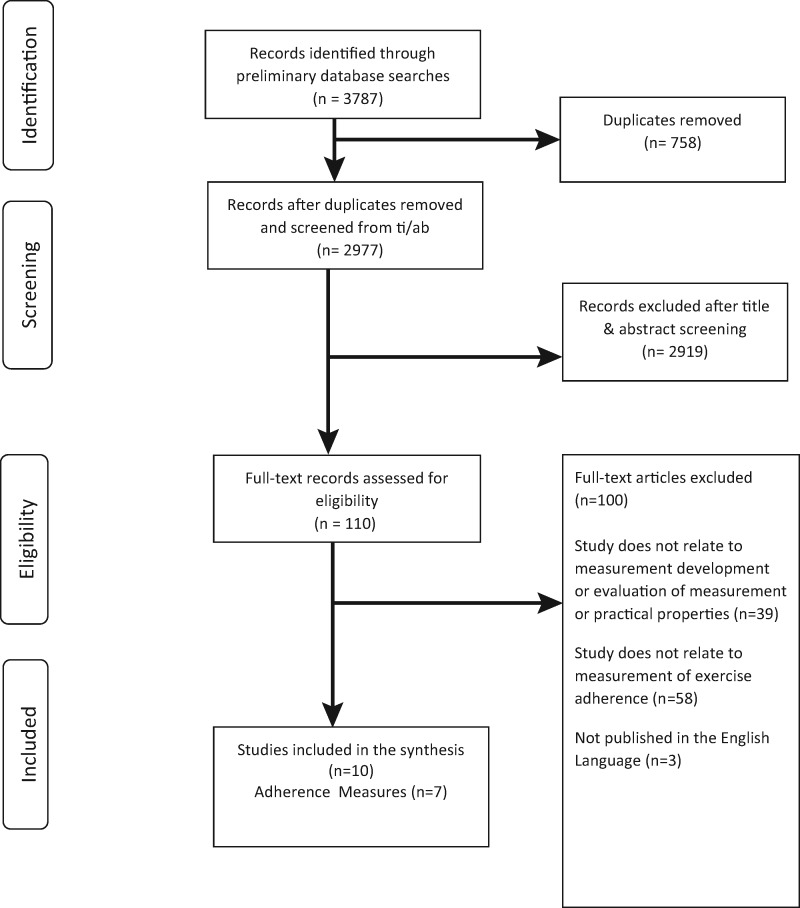
PRISMA flowchart for phase 2 of the systematic review

Evidence for measurement and/or practical properties were sought for the 49 reproducible measures identified in phase 1. Following removal of duplicates, 2977 records were identified. Following title and abstract screening, 110 full-text articles were retrieved and reviewed in full and 10 retained for phase 2 (Fig. 2) [22, 40–48].

These 10 articles provide evidence for seven clearly defined measures of exercise adherence in an MSK population. Three are clinician-reported: Hopkins Rehabilitation Engagement Rating Scale (HRERS) [41], Pittsburgh Rehabilitation Participation Scale (PRPS) [42] and the Sport Injury Rehabilitation Adherence Scale (SIRAS) [45]. Four are patient-reported: Adherence to Exercise Scale for Older Patients (AESOP) [22], Community Healthy Activities Model Program for Seniors Activities Questionnaire for Older Adults (CHAMPS) [44]; the Modified Rehabilitation Adherence Questionnaire (RAQ-M) [42]; and the Rehabilitation Overadherence Questionnaire (ROAQ) [48]. Attempts to contact measurement developers for further information were unsuccessful.

### Data extraction: inter-rater reliability

Evidence for 40/107 COSMIN items across 5/10 COSMIN domains (A, B, D, E, F) was extracted. Agreement exceeded 80% for only 20 items (50%) [31]. Disagreement was mainly due to poor reporting of evidence in the reviewed papers, associated interpretation difficulties, reading errors or difficulties applying the checklist.

### Study characteristics

Although six studies were adequately sized for evaluative purposes (range 145–249) [34], four included fewer than 100 patients [22, 40, 45, 47]. The ages of patients ranged from 13 to 96 years (see Table 1). Studies covered a wide range of MSK settings: athletes with acquired knee injuries [43, 45–47]; general MSK disorders in outpatient settings [40]; older patients with generalized MSK conditions [22, 42, 44]; acute inpatient populations [41]; and athletic adolescents with MSK injuries [48].

**Table 1:** Characteristics of reviewed measures used to assess exercise adherence in patients with MSK problems

Measure (developer, year)	Evaluations (n)	Construct	Domains (items)	Response options	Recall	Score range	Admin (time)
Clinician-completed							
Hopkins Rehabilitation Engagement Rating Scale (HRERS)	1	Behavioural observations of patients during acute inpatient rehabilitation	Five items: Attendance at rehabilitation session (1) Frequency of required verbal/physical prompts (1) Perceived positive attitude to exercise (2) Perceived need for and benefit from rehabilitative exercise Active participation in rehabilitative exercise (1)	Six-point descriptive: Never (1) Seldom (2) Some of the time (3) Most of the time (4) Nearly always (5) Always (6)	At the time of the rehabilitation session/at time of discharge to represent a summary of observations during patients’ inpatient stay	Simple summation: range 5–30, where 5 is poor and 30 is best engagement in the therapy process	NR
(Kortte *et al.*, 2007) [41]							
Pittsburgh Rehabilitation Participation Scale (PRPS)	1	Observed patient ‘participation’ in a therapy session	Single item to assess patient participation in a therapy session	Detailed 6-point Likert scale, ranging from: None (1): patient refused entire session or did not participate in exercises to Excellent (6): patient participated in all exercises with max effort, finished all exercises and actively took interest in exercises and/or future therapy sessions	At the time of the rehabilitation session	One response is selected: range 1 (poor) to 6 excellent participation	NR
(Lenze *et al.*, 2004) [42]							
Sport Injury Rehabilitation Adherence Scale (SIRAS)	8	Adherence during rehabilitation sessions	Three items: Perceived intensity/effort/exertion (1) Frequency of following therapist instructions (1) Receptive to change in rehabilitation exercise (1)	Five-point numerical rating scale:Anchors: Minimum effort (1) to maximum effort (5) Never (1) to always (5) Very unreceptive (1) to very receptive (5)	1 week	Index (composite) score—summation of score for the three items: range 0–15, where 1 is lower adherence, and 15 is maximal adherence	NR
(Brewer *et al.* 1999) [45]							
Patient-completed							
Adherence to Exercise Scale for Older Patients (AESOP)	1	Social cognitive theory constructs for predicting home exercise programme (HEP) adherence in older adults: self-efficacy expectations, outcome expectations and outcome expectancies	42 items: Self-efficacy expectations (15) Outcome expectations (16) Outcome expectancies (11)	Five-point agreement: Strongly disagree (1) Disagree (2) No opinion (3) Agree (4) Strongly agree (5)	2 weeks	Simple summation: Range 15–75 Range 16–80 Range 11–55 Lower scores suggest lower levels of adherence	NR
(Hardage *et al.* 2007) [22]							
Community Healthy Activities Model Program for Seniors (CHAMPS)	1	Types and intensity levels of physical activity	41 items: Ranging over activities of daily living, work related activities, social activities and leisure activities	Five-point agreement: Strongly disagree (1) Disagree (2) No opinion (3) Agree (4) Strongly agree (5)	4 weeks	Frequency of activities per week: number of minutes of physical activity per week across all activities Calorie expenditure: per week multiply esti mated duration of each activity by the MET value and summing across all activities Both can be calculated for: (i) Moderate and greater activity measures (ii) All activity measures Therefore, four scores possible	NR
(Stewart *et al.* 2001) [44]							
Modified Rehabilitation Adherence Questionnaire (RAQ-M)	1	Rehabilitation adherence in injured athletes	25 items: Perceived exertion (3) Pain tolerance (5) Self-motivation (5) Support from significant others (5) Scheduling (4) Environmental conditions (3)	Four-point agreement: Strongly disagree (1) Disagree (2) Agree (3) Strongly agree (4)	1 week	Simple item summation for each domain: Range 3–12 Range 5–20 Range 5–20 Range 5–20 Range 4–16 Range 3–12 Higher scores reflect greater levels of adherence	NR
(Shin *et al.* 2010) [43]							
Rehabilitation Overadherence Questionnaire (ROAQ)	2	Assessment of overadherence behaviours and beliefs in injured athletes	Two domains (10 items): Ignoring practitioner recommendations (6) Attempting an expe dited rehabilitation (4)	Five-point agreement: Never or strongly disagree (1) to Always or strongly agree (5)	NR	NR	NR
(Podlog *et al.* 2013) [48]							

n: number of studies evaluating the measurement and practical properties of each measure; NR: not reported; MET: metabolic energy equivalent.

### Adherence measures

Six of the seven measures were originally developed as measures of exercise adherence, including the following: sports injury rehabilitation (SIRAS, RAQ-M, ROAQ); acute MSK inpatient rehabilitation (HRERS, PRPS); and MSK home exercise programmes (AESOP). Although originally developed as a measure of physical activity, the CHAMPS has subsequently been evaluated as a measure of exercise adherence and hence is included in this review [43]. With the exception of the RAQ-M which was evaluated in Korean athletes, all measures were developed and evaluated in the USA. The characteristics and measurement properties of all reviewed measures are summarized in Tables 1 and 2 and Supplementary Tables S2 and S3, available at *Rheumatology* Online. Study methodological quality and the qualitative synthesis is summarized in Table 3.

**Table 2:** Methodological quality and investigated measurement and practical properties per measure per reviewed article

Article (n = 9)	Population (n)	Age mean (sd), years; range	Measures	Reliability		Validity			Responsiveness
				Internal reliability	Test–retest	Convergent/ divergent	Known groups	Structural	Responsiveness
Brewer *et al.* 2002 [47] Study 1	43 (practitioners)	Range 20–43	SIRAS	—	Poor	—	Poor	—	—
Study 2	12 (rehab patients)	29.33 (11.44)	SIRAS	—	Poor	—	—	—	—
Brewer *et al.* 2000 [46] Study 1	145 (orthopaedic outpatients)	43.95 (15.54)	SIRAS	Fair	—	Poor	—	Fair	—
Study 2	31 (sport related knee injury)	NR	SIRAS	—	Fair	—	—	—	—
Study 3	43 (rehab post-ACL repair)	NR	SIRAS	—	Fair	—	—	—	—
Brewer *et al.* 1999 [45]	31	NR	RAQ	Poor	Poor	Poor	—	—	—
			SIRAS						
Hardage *et al.* 2007 [22]	50	79.9; range 65–91	AESOP	—	Poor	Poor	Poor	—	—
			SF-12 mMSE GDS						
Kolt *et al.* 2007 [40] Study1	60 (physiotherapists = raters)	NR	SIRAS	—	Poor	—	Poor	Poor	—
Study 2	45 patients (general MSK)	>18 years	SIRAS	—	Poor	—	—	Poor	—
Kortte *et al.* 2007 [41]	208	56.7 (17.52); range 18–91	HRERS	Poor	Poor	Fair	Fair	Fair	—
			FIM BSI L-DIQ PANAS CHART						
Lenze *et al.* 2004 [42]	242	70.8 (14.8); range 20–96	PRPS	—	Fair	Poor	—	—	Poor
			FIM-motor						
Podlog *et al.* 2013 [48] Study 1	118 injured adolescent athletes	16.0 (1.4); range 13–18	RAOQ	Fair	—	Fair	—	Fair	—
			SPSQ AIMS I-PRRS						
Study 2	105 injured collegiate athletes	NR	RAOQ	Fair	—	Fair	—	Fair	—
			SPSQ AIMS I-PRRS						
Shin *et al.*, 2010 [43]	240 injured athletes	NR	RAQ-M	Fair	Poor	Poor	Poor	Poor	—
			SIRAS						
Stewart *et al.* 2001 [44]	249	74.1; range 65–90	CHAMPS	—	Good	Good	Good	—	Fair
			BMI SF-36 domains SPPB 6-min walk						

n: population size in included study; NR: not reported; 6-min walk: six-minute walking test; BSI: Brief Symptom Inventory; CHART: Craig Handicap Assessment and Reporting Technique; FIM: Functional Impact Measure; GDS: Geriatric Depression Scale; L-DIQ: Levine's Denial of Illness Questionnaire; mMSE: mini-Mental State Examination; PANAS: Positive and Affective Negative State; SF-12: Short-Form 12-item Health Survey; SF-36: Short-Form 36-item Health Survey; SPPB: Short Physical Performance Battery; HRERS: Hopkins Rehabilitation Engagement Rating Scale; PRPS: Pittsburgh Rehabilitation Participation Scale; SIRAS: Sport Injury Rehabilitation Adherence Scale; AESOP: Adherence to Exercise Scale for Older Patients; CHAMPS: Community Healthy Activities Model Program for Seniors; RAQ-M: Modified Rehabilitation Adherence Questionnaire; SPSQ: Self-Presentation in Sport Questionnaire; AIMS: Athletic Identity Measurement Scale; ROAQ: Rehabilitation Overadherence Questionnaire; I-PRRS: Modified Injury Psychological Readiness to Return to Sport Scale.

**Table 3:** Overall quality of measurement properties per reviewed measure of exercise adherence for MSK populations

Measure	Evaluations (n)	Reliability			Validity				Responsiveness
		Test–retest (intra/inter)	Internal consistency	Measurement error	Content	Convergent/ divergent	Known groups	Structural	Responsiveness
Therapist-completed									
_HRERS_	1	+limited	+limited	Nil	Nil	+limited	+limited	+limited	Nil
_PRPS_	1	+limited	Nil	Nil	Nil	+limited	Nil	Nil	−limited
_SIRAS_	8	+limited	+limited	Nil	Nil	+limited	+limited	Nil	Nil
Patient-completed									
_AESOP_	1	−limited	Nil	Nil	Nil	+limited	?limited	Nil	Nil
_CHAMPS_	1	−limited	Nil	Nil	Nil	+limited	?limited	Nil	−limited
_RAQ-M_	1	+limited	+limited	Nil	Nil	?limited	?limited	+limited	Nil
_ROAQ_	2	Nil	+limited	Nil	−limited	+limited	Nil	+limited	Nil

n: number of studies evaluating the measurement and practical properties of each measure; the overall quality of a measurement property is reported as adequate (+), not adequate (−), conflicting (±), or unclear (?); levels of evidence for the overall quality of each measurement property is strong, moderate, limited, conflicting, or unknown evidence. HRERS: Hopkins Rehabilitation Engagement Rating Scale; PRPS: Pittsburgh Rehabilitation Participation Scale; SIRAS: Sport Injury Rehabilitation Adherence Scale; AESOP: Adherence to Exercise Scale for Older Patients; CHAMPS: Community Healthy Activities Model Program for Seniors; RAQ-M: Modified Rehabilitation Adherence Questionnaire; ROAQ: Rehabilitation Overadherence Questionnaire.

#### Clinician-reported

The five-item HRERS assesses the therapist’s perception of an individual’s engagement in acute inpatient rehabilitation. There is limited evidence of reliability and validity following completion in a population of patients with spinal cord injury, stroke, amputation or hip/knee replacement [41]. The unidimensional structure (structural validity) of the HRERS as a measure of engagement was supported by principal component factor analysis across the different diagnostic groups. A high level of internal consistency for this single dimension (Cronbach’s α = 0.91) and acceptable inter-rater agreement (intraclass correlation coefficient (ICC) = 0.73) was reported [41]. Evidence of known-groups validity was provided against groups defined by a range of external criteria hypothesized to be associated with engagement including scores on the Functional Impact Measures (FIM) and rates of therapy absenteeism. Small correlations were reported between the HRERS and a range of clinical variables including depression (r = 0.24), denial of illness (r = 0.30), self-rated negative affect (r *=* −0.23) and level of functioning (r = 0.22) [41]; although the authors suggest that hypothesized associations were supported, these were not clearly stated, hence limiting interpretation in support of measurement validity.

The single-item PRPS is used to rate patient participation during each treatment session of acute inpatient rehabilitation [42]. Item development involved therapist interviews and therapy session observation of older patients with generalized MSK problems. There is limited evidence of reliability and validity following completion with older people with generalized MSK conditions [42]. High values of inter-rater reliability (range of ICC = 0.91–0.96) were reported [42]. Small correlations between the PRPS and the FIM-motor (range r = 0.38), with change in FIM-motor (r = 0.32) and length of stay were reported (r = −0.13; P < 0.05) (Supplementary Table S3) [41]; however, the absence of *a priori* hypothesized associations between variables limits interpretation. Similarly, although a statistically significant score improvement was reported in those inpatients with a length of stay >9 days [score increase from 4.29 ± 0.93 to 4.67 ± 1.04; p < 0.001], external anchors against which change in participation may be judged or suggestions for interpretation of score change are not provided.

The three-item SIRAS is used by therapists to rate the degree to which patients exert themselves, follow the practitioner’s instructions and advice, and are receptive to changes in the rehabilitation programme during a given rehabilitation session. The single factor structure of the SIRAS (exercise adherence) is supported by several studies following completion by athletes and the general MSK population [40, 46]. Internal consistency evaluations further support reporting the SIRAS as a single index value [46]. Acceptable levels of internal consistency supports application in groups of patients (Cronbach’s α range 0.82–0.8) [46, 47]. Poor to high levels of inter-rater (ICC range = 0.57–0.77; Rater Agreement Index range = 0.84–0.94) and acceptable 1-week test–retest reliability has been reported (range = 0.63–0.77) [39, 45]. Evidence in support of known-groups validity is provided following the assessment of standardized vignettes describing three levels of adherence in athletes [40, 47].

#### Patient-reported measures

The AESOP is a 42-item interview-administered questionnaire, developed to assess exercise adherence in older patients [22]. The measure constitutes three domains, informed by social cognitive theory: self-efficacy expectations (15 items), outcome expectations (16 items) and outcome expectancies (11 items). Although acceptable test–retest reliability was reported for two domains—self-efficacy expectations (ICC = 0.80) and outcome expectations (ICC = 0.77)—low levels were reported for outcome expectancies (ICC = 0.33) [22]. All correlations between the three AESOP domains and the Short Form 12-item Health Survey (SF-12, version 2) physical and mental component scales were very small; the absence of *a priori* hypothesized associations between variables limits interpretation in support of measurement validity.

The CHAMPS activities questionnaire is a 41-item patient-reported or interview-administered questionnaire. The CHAMPS is a measure of physical activity that has been evaluated for use as a measure of exercise adherence in daily life [44]. The CHAMPS asks about activities that you may have done in the past 4 weeks. The information is used to calculate frequency of activities—the number of minutes of physical activity per week and the calories expended per week in all physical activities. Each score can be calculated for moderate and greater activity levels, and all activity levels. Hence, four scores are possible. Data from an intervention trial to increase activity levels among community-dwelling older people (CHAMPS trial) was assessed for score stability at 6 months (for participants in the non-active treatment or control group and hence not expected to change) and 2-week test–retest reliability [43]. Moderate levels of test–retest reliability were reported across the different CHAMP scores (range = 0.58–0.67); the authors suggest that the low levels could be influenced by the difficulty in recalling activities. As hypothesized, patients who were classified as being inactive had significantly lower CHAMPS scores when compared with more active patients (P < 0.001) [44]. Correlations between the CHAMPS scores and a range of health measures supported *a priori* stated hypotheses, providing acceptable evidence in support of the CHAMPS as a measure of physical activity in older people. Evidence suggests that the CHAMPS can detect improvement in physical activity levels in a large group of participants receiving an active intervention to facilitate increased activity. These changes were greater for the frequency measures [effect size = 0.54 and 0.64) when compared with the change in caloric expenditure (effect size = 0.38 and 0.42), suggesting moderate levels of responsiveness.

The 25-item RAQ-M was developed to evaluate exercise adherence in injured athletes [43]. The original 40-item RAQ developed by Fisher [48] was excluded from phase 1 of the review due to insufficient information to support reproduction. Moreover, evidence of poor reliability and validity have underpinned recommendations for significant redevelopment [45]. The RAQ-M includes six domains of adherence: perceived exertion (three items), pain tolerance during exercise (five items), self-motivation (five items), support from significant others (five items), scheduling (four items) and environmental conditions (three items). The revised six-domain structure was informed by an exploratory and subsequent confirmatory factor analysis [43]. An initial analysis of the internal consistency reliability of the six domains ranged from 0.66 (perceived exhaustion) to 0.87 (scheduling). Acceptable 2-week test–retest reliability values were reported, and ranged from 0.64 (pain tolerance) to 0.81 (support from significant others); however, the relative stability of these athletes’ injuries was not reported. Small to moderate levels of association were reported between the RAQ-M domains and three adherence measures, including the SIRAS [43]; however, the absence of *a priori* hypothesized associations between variables limits interpretation. A process of forward and backward translation facilitated translation of the measure from English into Korean.

The 10-item ROAQ purports to measure the tendency for an athlete to be overly adherent to a rehabilitation regime, ignore practitioner recommendations and attempt an expedited rehabilitation and return to sport [48]. Items were generated following a review of the literature for indicators of over-adherence and discussion with experts in sports psychology and clinical rehabilitation of athletes. Young athletes were not consulted. The two-domain factor structure was supported following ROAQ completion by two independent groups of athletes, the first aged 13–18 years (study 1) and the second older adolescents (study 2). Acceptable levels of internal consistency reliability (α >0.70) were reported for both domains in both groups. There is limited evidence in support of the construct validity of the measure; the absence of *a priori* hypothesized associations between variables limits interpretation. The ROAQ has only been evaluated by the developers.

## Discussion

Despite the large number of reported approaches to assessing exercise adherence, clear recommendations for the assessment of exercise adherence in MSK populations cannot be made because of poor reporting, inadequate quality and meagre conceptual underpinnings of reviewed measures. Evidence for the seven short-listed measures was mostly limited or not available. Although originally developed as a measure of physical activity in older adults, the CHAMPS has been applied and evaluated as a measure of exercise adherence [44]. Application of a measure for a purpose other than that for which it was developed undermines the validity of the results and limits meaningful interpretation with which to inform decision-making.

Evidence of measurement error, content or face validity, data quality, precision and score interpretation was not identified for any of the reviewed measures. None of the studies explored the relevance, acceptability or appropriateness of measures to the target population, or considered respondent burden. Although all measures had limited evidence of construct validity (convergent; known groups), the absence of *a priori* hypothesized associations between variables limits interpretation and undermines the quality of evidence [34]. Only three measures had limited evidence of structural validity; and just two had (poor) evidence describing measurement responsiveness. There was no evidence of involvement of patients as research partners during the development of any measure. This is a finding reported in other reviews [32, 37], but increasingly viewed as an important consideration in enhancing the relevance and validity of patient-centred outcome assessment [50–52]. Only four of the reviewed measures were patient-reported; the additional measures were clinician-reported. Discrepancies between patients and health professionals with regards to understanding or defining a good outcome have been widely reported [53–56]. It is likely that patients and clinicians have different views about what constitutes adherence. A collaborative exploration of the views of stakeholders, including patients, health professionals and rehabilitation experts, with regards to what should be assessed, by whom, when and in what context is essential to the development of assessment in this field. A new patient-derived measure with a clear conceptual underpinning that reflects the needs of key stakeholders is essential to ensure meaningful investigation of the challenges and burden of adhering to exercise [52].

The review is strengthened by use of the Preferred Reporting Items for Systematic Reviews and Meta-Analyses guidelines [32]. The methodological and quality concerns highlighted by the review were underpinned by a transparent evaluation of study (COSMIN) and measurement quality [33–35]. This is the first study to evaluate the intra-rater reliability of the COSMIN four-point check-list: poor intra-reviewer agreement between trained, but relatively inexperienced, reviewers was found. These findings highlight the challenge for reviewers of patient reported outcome measure (PROM) quality: poor quality reporting often fails to match the rigors of the COSMIN gold standard checklist and inexperienced reviewers may struggle to unpack complicated or poor quality papers. We recommend that all reviews include an experienced reviewer to guide extraction and/or act as arbiter. Moreover, clear guidance for transparent reporting of PROM quality in published papers is required.

Our extensive search strategy utilized multiple major databases and although limited to English-language publications, English-language abstracts for non-English publications were reviewed and, with the exception of three articles excluded due to language, were excluded due to irrelevance. It is unlikely that any selection bias resulted. The focus of our review was adults with MSK conditions, and our results are not necessarily applicable to non-MSK populations.

A recent review of self-report measures of exercise adherence completed by patients with long-term health problems and undertaking unsupervised home-based exercise programmes similarly concluded that measures are largely unreproducible with extremely limited evidence of essential psychometric properties, thus preventing any clear recommendations for assessment [29]. Another review related to home exercise adherence concluded that there were no valid measures of home exercise adherence for chronic low back pain [57]. The lack of good quality measures and transparency in adherence reporting highlighted in these review must be addressed [29, 57]. In our review only 15% (7 from 45) of the measures purportedly used to assess exercise adherence were taken forward from phases 1 to 2 of the review due to inadequate detail or lack of supporting reference. Appropriate reporting of assessment approaches is essential to ensuring that adherence data are appropriately utilized. Moreover, good reporting contributes to the evidence base with which to inform measurement selection. The Consolidated Standards of Reporting Trials statement [58, 59], and recent patient-reported outcome extension seek to encourage more complete and transparent reporting of assessment approaches and outcome data [60].

In conclusion, we cannot recommend any measure of exercise adherence for MSK settings due to the inadequacy of essential measurement and practical properties for clearly defined measures. Our review provides a critical insight into the many failings of published measures of exercise adherence, though this may reflect the difficulty of measuring adherence. In particular, the conceptual underpinnings of what should be assessed, by whom, when and in what context are poorly considered and essential for future research. Moreover, the transparency in adherence measure reporting must be improved.

*Funding*: This work was supported by the Chartered Society of Physiotherapy Charitable Trust [grant number PRF/12/13].

*Disclosure statement*: The authors have declared no conflicts of interest.

## Supplementary data

Supplementary data are available at *Rheumatology* Online.

## Supplementary Material

Supplementary Data
