# Supplementary material for: Quality and acceptability of measures of exercise adherence in musculoskeletal settings: a systematic review
Source: Rheumatology (Oxford). 2016 Dec 23;56(3):426–38. doi: 10.1093/rheumatology/kew422 (PMC5410983; doi:10.1093/rheumatology/kew422)
Supplement: Supplementary Data [file kew422_Supp.docx]

**SUPPLEMANTARY DATA**

**Search strategy for phase 1**

The search strategies used title/abstract words and relevant indexing to capture the concept of exercise adherence in the context of musculoskeletal rehabilitation, for adult patients. The strategies also contained the following exclusions: cardiac rehabilitation, pulmonary rehabilitation, neuro* rehabilitation, stroke.

To capture exercise adherence: search terms/synonyms for adherence [see below] were searched in proximity (within 3 words) to terms for exercise, in the title/abstract fields; secondly, search terms/synonyms for adherence were searched in combination (AND) with database subject headings for exercise/therapeutic exercise etc; thirdly, search terms/synonyms for exercise were searched in combination (AND) with database subject headings for patient compliance; finally, the database subject headings for exercise/therapeutic exercise etc. were searched in combination (AND) with database subject headings for patient compliance.

To capture musculosekeletal rehabilitation, the above searches were combined (AND) with the search terms/synonyms in the title/abstract fields and the database headings listed below.

**Adherence terms:** adher*, nonadher*, complian*, noncomplian*, concordan*, cooperat*, co-operat*, uncooperat*, unco-operat*, engag*, disengag*, behaviour#, behavior#, MeSH: "Patient Compliance"

**Exercise terms:** activ*, exercis*, physical n3 train*, weight n3 train*, sport#, rehab*, MeSH: "Therapeutic Exercise+", "Exercise Therapy+", "Exercise+", "Physical Activity", "Motor Activity"

**Musculoskeletal rehabilitation terms:** osteopath*, chiropract*, musculoskeletal, msk, physiotherap*, rehabilitat*, osteoarthrit*, spondyl* , osteitis , osteochondritis, arthropathy, bursitis,"shoulder impingement" , myalgia, lordosis, sacroiliac, sciatica, cervicogenic, dyskinesis, tendinitis, tendinopathy, allodynia, hyperalgesia, subluxation, disc , misalignment, "osteopathic lesion" , "frozen shoulder" , "degenerative joint disease", muscular n3 pain, back n3 pain, lumbar n3 pain, lumbo* n3 pain, spine n3 pain, spinal n3 pain, neck n3 pain, cervical n3 pain, knee* n3 pain, hips n3 pain, hip n3 pain, shoulder n3 pain, ankle# n3 pain, foot n3 pain, feet n3 pain, elbow# n3 pain, hand# n3 pain, "flank pain", "buttock pain", "joint pain", "radicular pain", neuralgia, lumbago, arthralgia, "adverse neural tension", "muscle tear#", sprain* n5 musc*, strain* n5 musc*, MesH: "Osteopathy", "Osteopathic Medicine", "Chiropractic", "Manipulation, Chiropractic", "Musculoskeletal Diseases+", "Sciatica", "Tendinopathy+", "Allodynia", "Hyperalgesia", "Subluxation", "Back Pain+", "Neck Pain", "Neuralgia+", "Elbow Pain", "Arthralgia+", ("Musculoskeletal System+" AND "Pain+")

**Indicative search strategy (Medline/CINAHL Plus with Fulltext, via EBSCOHost)**

| # | Query |
| --- | --- |
| S1 | TI ( (adher* or nonadher*) n3 (activ* or exercis* or (physical n3 train*) or (weight n3 train*) or sport# or rehab*) ) OR AB ( (adher* or nonadher*) n3 (activ* or exercis* or (physical n3 train*) or (weight n3 train*) or sport# or rehab*) ) |
| S2 | TI ( (complian* or noncomplian*) n3 (activ* or exercis* or (physical n3 train*) or (weight n3 train*) or sport# or rehab*) ) OR AB ( (complian* or noncomplian*) n3 (activ* or exercis* or (physical n3 train*) or (weight n3 train*) or sport# or rehab*) ) |
| S3 | TI (concordan* n3 (activ* or exercis* or (physical n3 train*) or (weight n3 train*) or sport# or rehab*) ) OR AB (concordan* n3 (activ* or exercis* or (physical n3 train*) or (weight n3 train*) or sport# or rehab*) ) |
| S4 | TI ( (cooperat* or co-operat* or uncooperat* or unco-operat*) n3 (activ* or exercis* or (physical n3 train*) or (weight n3 train*) or sport# or rehab*) ) OR AB ( (cooperat* or co-operat* or uncooperat* or unco-operat*) n3 (activ* or exercis* or (physical n3 train*) or (weight n3 train*) or sport# or rehab*) ) |
| S5 | TI ( (engag* or disengag*) n3 (activ* or exercis* or (physical n3 train*) or (weight n3 train*) or sport# or rehab*) ) OR AB ( (engag* or disengag*) n3 (activ* or exercis* or (physical n3 train*) or (weight n3 train*) or sport# or rehab*) ) |
| S6 | TI ( (behaviour# or behavior#) n3 (activ* or exercis* or (physical n3 train*) or (weight n3 train*) or sport# or rehab*) ) OR AB ( (behaviour# or behavior#) n3 (activ* or exercis* or (physical n3 train*) or (weight n3 train*) or sport# or rehab*) ) |
| S7 | S1 OR S2 OR S3 OR S4 OR S5 OR S6 |
| S8 | TI ( adher* or nonadher* ) OR AB ( adher* or nonadher* ) |
| S9 | TI ( complian* or noncomplian* ) OR AB ( complian* or noncomplian* ) |
| S10 | TI (concordan*) OR AB (concordan*) |
| S11 | TI ( cooperat* or co-operat* or uncooperat* or unco-operat* ) OR AB ( cooperat* or co-operat* or uncooperat* or unco-operat* ) |
| S12 | TI ( engag* or disengag* ) OR AB ( engag* or disengag* ) |
| S13 | TI ( behaviour# or behavior# ) OR AB ( behaviour# or behavior# ) |
| S14 | S8 OR S9 OR S10 OR S11 OR S12 OR S13 |
| S15 | (MH "Therapeutic Exercise+") OR (MH "Exercise Therapy+") |
| S16 | (MH "Exercise+") |
| S17 | (MH "Physical Activity") or (MH "Motor Activity") |
| S18 | S15 OR S16 OR S17 |
| S19 | S14 AND S18 |
| S20 | TI ( (activ* or exercis* or (physical n3 train*) or (weight n3 train*) or sport# or rehab*) ) OR AB ( (activ* or exercis* or (physical n3 train*) or (weight n3 train*) or sport# or rehab*) ) |
| S21 | (MH "Patient Compliance") |
| S22 | S20 AND S21 |
| S23 | S18 AND S21 |
| S24 | S7 OR S19 OR S22 OR S23 |
| S25 | (MH "Physical Therapy+") or (MH "Exercise Movement Techniques+") OR (MH "Exercise Therapy+") |
| S26 | MH ("Osteopathy") OR (MH "Osteopathic Medicine") |
| S27 | (MH "Chiropractic") OR (MH "Manipulation, Chiropractic") |
| S28 | TI ( osteopath* or chiropract* or musculoskeletal or msk ) OR AB ( osteopath* or chiropract* or musculoskeletal or msk ) |
| S29 | TI ( physiotherap* or rehabilitat* ) OR AB ( physiotherap* or rehabilitat* ) |
| S30 | (MH "Musculoskeletal Diseases+") |
| S31 | TI ( osteoarthrit* or spondyl* or osteitis or osteochondritis ) OR AB ( osteoarthrit* or spondyl* or osteitis or osteochondritis ) |
| S32 | TI ( arthropathy or bursitis or "shoulder impingement" or myalgia ) OR AB ( arthropathy or bursitis or "shoulder impingement" or myalgia ) |
| S33 | TI ( lordosis or sacroiliac or sciatica or cervicogenic ) OR AB ( lordosis or sacroiliac or sciatica or cervicogenic ) |
| S34 | (MH "Sciatica") |
| S35 | (MH "Tendinopathy+") |
| S36 | (MH "Allodynia") |
| S37 | TI ( dyskinesis or tendinitis or tendinopathy or allodynia ) OR AB ( dyskinesis or tendinitis or tendinopathy or allodynia ) |
| S38 | (MH "Hyperalgesia") |
| S39 | (MH "Subluxation") |
| S40 | TI ( hyperalgesia or subluxation or disc or misalignment ) OR AB ( hyperalgesia or subluxation or disc or misalignment ) |
| S41 | TI ( "osteopathic lesion" or "frozen shoulder" or "degenerative joint disease" ) OR AB ( "osteopathic lesion" or "frozen shoulder" or "degenerative joint disease" ) |
| S42 | TI muscular n3 pain OR AB muscular n3 pain |
| S43 | TI ( (back or lumbar or lumbo* or spine or spinal) n3 pain ) OR AB ( (back or lumbar or lumbo* or spine or spinal) n3 pain ) |
| S44 | TI ( (neck or cervical) n3 pain ) OR AB ( (neck or cervical) n3 pain ) |
| S45 | TI ( (knee* or hip or hips or shoulder*) n3 pain ) OR AB ( (knee* or hip or hips or shoulder*) n3 pain ) |
| S46 | TI ( (ankle# or foot or feet or elbow# or hand#) n3 pain ) OR AB ( (ankle# or foot or feet or elbow# or hand#) n3 pain ) |
| S47 | TI ( "flank pain" or "buttock pain" or "joint pain" or "radicular pain" ) OR AB ( "flank pain" or "buttock pain" or "joint pain" or "radicular pain" ) |
| S48 | (MH "Back Pain+") |
| S49 | (MH "Neck Pain") |
| S50 | (MH "Neuralgia+") |
| S51 | (MH "Elbow Pain") |
| S52 | (MH "Arthralgia+") |
| S53 | TI ( neuralgia or lumbago or arthralgia ) OR AB ( neuralgia or lumbago or arthralgia ) |
| S54 | TI ( "adverse neural tension" or "muscle tear#" ) OR AB ( "adverse neural tension" or "muscle tear#" ) |
| S55 | TI ( (sprain* or strain*) n5 musc* ) OR AB ( (sprain* or strain*) n5 musc* ) |
| S56 | (MH "Musculoskeletal System+") |
| S57 | (MS "Pain+") |
| S58 | S56 AND S57 |
| S59 | S25 OR S26 OR S27 OR S28 OR S29 OR S30 OR S31 OR S32 OR S33 OR S34 OR S35 OR S36 OR S37 OR S38 OR S39 OR S40 OR S41 OR S42 OR S43 OR S44 OR S45 OR S46 OR S47 OR S48 OR S49 OR S50 OR S51 OR S52 OR S53 OR S54 OR S55 OR S58 |
| S60 | TI ( "cardiac rehabilitation" or "pulmonary rehabilitation" or "neuro* rehabilitation" or stroke or cancer or carcinoma ) OR AB ( "cardiac rehabilitation" or "pulmonary rehabilitation" or "neuro* rehabilitation" or stroke or cancer) |
| S61 | TI ( child* NOT adult* ) OR AB ( child* NOT adult* ) |
| S62 | TI ( infan* NOT adult* ) OR AB ( infan* NOT adult* ) |
| S63 | (MH "Child+") NOT (MH "Adult+") |
| S64 | S60 OR S61 OR S62 OR S63 |
| S65 | S59 not S64 |
| S66 | S24 AND S65 |

**Search strategy for phase 2**

For each shortlisted named measure, the name was searched as a word/phrase in the title/abstract fields of each database. Where the results set exceeded 50 records, the Sensitive search filter for measurement properties found in Appendix 2 of Terwee et al. (2009) [1] was additionally applied.

**Search strategy for measurement properties filter (Medline/CINAHL Plus with Fulltext, via EBSCOHost)**

| S1 | (MH "Methods") |
| --- | --- |
| S2 | Validation Studies |
| S3 | Comparative Study |
| S4 | (MH "Psychometrics") |
| S5 | TI psychometr* OR AB psychometr* |
| S6 | TI ( clinimetr* OR clinometr* ) OR AB ( clinimetr* OR clinometr* ) |
| S7 | (MH "Outcome Assessment (Health Care)") |
| S8 | TI "outcome assessment" OR AB "outcome assessment" |
| S9 | TX "outcome measure*" |
| S10 | (MH "Observer Variation") |
| S11 | TI "observer variation" OR AB "observer variation" |
| S12 | (MH "Health Status Indicators") |
| S13 | (MH "Reproducibility of Results") |
| S14 | TI reproducib* OR AB reproducib* |
| S15 | (MH "Discriminant Analysis") |
| S16 | TI ( reliab* OR unreliab* ) OR AB ( reliab* OR unreliab* ) |
| S17 | TI valid* OR AB valid* |
| S18 | TI coefficient OR AB coefficient |
| S19 | TI ( homogeneity OR homogeneous ) OR AB ( homogeneity OR homogeneous ) |
| S20 | TI "internal consistency" OR AB "internal consistency" |
| S21 | TI ( cronbach* AND (alpha OR alphas) ) OR AB ( cronbach* AND (alpha OR alphas) ) |
| S22 | TI ( item AND (correlation* OR selection* OR reduction*) ) OR AB ( item AND (correlation* OR selection* OR reduction*) ) |
| S23 | TI agreement OR AB agreement |
| S24 | TI ( precision OR imprecision ) OR AB ( precision OR imprecision ) |
| S25 | TI "precise values" OR AB "precise values" |
| S26 | TI test-retest OR AB test-retest |
| S27 | TI ( test AND retest ) OR AB ( test AND retest ) |
| S28 | TI ( reliab* AND (test OR retest) ) OR AB ( reliab* AND (test OR retest) ) |
| S29 | TI stability OR AB stability |
| S30 | TI ( interrater OR inter-rater ) OR AB ( interrater OR inter-rater ) |
| S31 | TI ( intrarater OR intra-rater ) OR AB ( intrarater OR intra-rater ) |
| S32 | TI ( intertester OR inter-tester ) OR AB ( intertester OR inter-tester ) |
| S33 | TI ( intratester OR intra-tester ) OR AB ( intratester OR intra-tester ) |
| S34 | TI ( interobserver OR inter-observer ) OR AB ( interobserver OR inter-observer ) |
| S35 | TI ( intraobserver OR intra-observer ) OR AB ( intraobserver OR intra-observer ) |
| S36 | TI ( intertechnician OR inter-technician ) OR AB ( intertechnician OR inter-technician ) |
| S37 | TI ( intratechnician OR intra-technician ) OR AB ( intratechnician OR intra-technician ) |
| S38 | TI ( interexaminer OR inter-examiner ) OR AB ( interexaminer OR inter-examiner ) |
| S39 | TI ( intraexaminer OR intra-examiner ) OR AB ( intraexaminer OR intra-examiner ) |
| S40 | TI ( interassay OR inter-assay ) OR AB ( interassay OR inter-assay ) |
| S41 | TI ( intraassay OR intra-assay ) OR AB ( intraassay OR intra-assay ) |
| S42 | S1 OR S2 OR S3 OR S4 OR S5 OR S6 OR S7 OR S8 OR S9 OR S10 OR S11 OR S12 OR S13 OR S14 OR S15 OR S16 OR S17 OR S18 OR S19 OR S20 OR S21 OR S22 OR S23 OR S24 OR S25 OR S26 OR S27 OR S28 OR S29 OR S30 OR S31 OR S32 OR S33 OR S34 OR S35 OR S36 OR S37 OR S38 OR S39 OR S40 OR S41 |
| S43 | TI ( interindividual OR inter-individual ) OR AB ( interindividual OR inter-individual ) |
| S44 | TI ( intraindividual OR intra-individual ) OR AB ( intraindividual OR intra-individual ) |
| S45 | TI ( interparticipant OR inter-participant ) OR AB ( interparticipant OR inter-participant ) |
| S46 | TI ( intraparticipant OR intra-participant ) OR AB ( intraparticipant OR intra-participant ) |
| S47 | TI ( kappa OR kappa's OR kappas ) OR AB ( kappa OR kappa's OR kappas ) |
| S48 | TI repeatab* OR AB repeatab* |
| S49 | TI ( replicab* AND (measure OR measures OR findings OR result OR results OR test OR tests) ) OR AB ( replicab* AND (measure OR measures OR findings OR result OR results OR test OR tests) ) |
| S50 | TI ( repeated AND (measure OR measures OR findings OR result OR results OR test OR tests) ) OR AB ( repeated AND (measure OR measures OR findings OR result OR results OR test OR tests) ) |
| S51 | TI ( generaliza* OR generalisa* ) OR AB ( generaliza* OR generalisa* ) |
| S52 | TI concordance OR AB concordance |
| S53 | TI ( intraclass AND correlation* ) OR AB ( intraclass AND correlation* ) |
| S54 | TI discriminative OR AB discriminative |
| S55 | TI "known group" OR AB "known group" |
| S56 | TI ( "factor analysis" OR "factor analyses" ) OR AB ( "factor analysis" OR "factor analyses" ) |
| S57 | TI dimension* OR AB dimension* |
| S58 | TI subscale* OR AB subscale* |
| S59 | TI ( multitrait AND scaling AND (analysis or analyses) ) OR AB ( multitrait AND scaling AND (analysis or analyses) ) |
| S60 | TI "item discriminant" OR AB "item discriminant" |
| S61 | TI "interscale correlation" OR AB "interscale correlation" |
| S62 | TI ( error OR errors ) OR AB ( error OR errors ) |
| S63 | TI "individual variability" OR AB "individual variability" |
| S64 | TI ( variability AND (analysis OR values) ) OR AB ( variability AND (analysis OR values) ) |
| S65 | TI ( uncertainty AND (measurement or measuring) ) OR AB ( uncertainty AND (measurement or measuring) ) |
| S66 | TI "standard error of measurement" OR AB "standard error of measurement" |
| S67 | TI sensitiv* OR AB sensitiv* |
| S68 | TI responsive* OR AB responsive* |
| S69 | TI ( (minimal OR minimally OR clinical OR clinically) AND (important OR significant OR detectable) AND (change OR difference) ) OR AB ( (minimal OR minimally OR clinical OR clinically) AND (important OR significant OR detectable) AND (change OR difference) ) |
| S70 | TI ( small* AND (real OR detectable) AND (change OR difference) ) OR AB ( small* AND (real OR detectable) AND (change OR difference) ) |
| S71 | TI "meaningful change" OR AB "meaningful change" |
| S72 | TI "ceiling effect" OR AB "ceiling effect" |
| S73 | TI "floor effect" OR AB "floor effect" |
| S74 | TI "item response model" OR AB "item response model" |
| S75 | TI IRT OR AB IRT |
| S76 | TI ( "differential item functioning" OR DIF ) OR AB ( "differential item functioning" OR DIF ) |
| S77 | TI Rasch OR AB Rasch |
| S78 | TI "computer adaptive testing" OR AB "computer adaptive testing" |
| S79 | TI "item bank" OR AB "item bank" |
| S80 | TI "cross-cultural equivalence" OR AB "cross-cultural equivalence" |

**Supplementary Table S1: Summary of all Exercise Adherence Measures**

| Measure | Developer | Primary purpose  (e.g. adherence, physical activity/ population etc) | Brief description of domains measures |
| --- | --- | --- | --- |
| The Rapid Assessment of Physical Activity (RAPA) | Topolski et al 2006 [2] | Amount and intensity of physical activity of older adult patients | 2 sections: 1 that tests aerobic activities and another for strength and flexibility. 9 yes/ no questions. |
| Stages of Exercise Change Questionnaire | Dannecker et al. 2003 [3] | Stages of change measure of the Trans-theoretical Model for exercise behavior. |  |
| Community Health Activities Model Program for Seniors (CHAMPS) | Stewart et al. 1998 [4] | Types and intensity levels of Physical activity | 41 items measuring activities of daily living, work –related, social activities and leisure activities |
| Behavioral Risk Factor Surveillance System | Remington et al. 1988 [5] | Health survey | Telephonic questionnaire with 20 core modules and 16 optional modules |
| Sport Injury Rehabilitation Adherence Scale (SIRAS) | Brewer et al 2000 [6] | Adherence during clinic-based rehabilitation programmes | 3 item measuring 1) Intensity of effort on rehabilitation exercise; 2) Frequency of following practitioner’s instructions and advice and 3) receptivity to changes in the physical therapy programme. |
| Short Questionnaire to Assess health enhancing physical activity (SQUASH) | Wendel-Vos et al. 2003 [7] | Walking and bicycling habits | Asks how many days per week walking and cycling activities were performed and how much time on average was engaged in this, and (if applicable) how strenuous this activity was. |
| Tegner activity scale | Tegner and Lysolm 1985 [8] | Activity level post Knee ligament injury | Indicate the HIGHEST level of activity that patient participated in BEFORE INJURY and the highest level that patient is able to participate in CURRENTLY |
| The Physical Activity Scale for the Elderly questionnaire (PASE) | Washburn et al. 1993 [9] | Physical activity questionnaire for elderly | Frequency and duration of leisure time, household and work related activity |
| Minnesota Leisure Time Physical Activity questionnaire | Periera et al. 1997 [10] | Leisure time activities | 2 sections: walking and miscellaneous and conditioning exercise. Yes or No for each activity |
| Yale Physical Activity Survey (YPAS) | Depietro et al. 1993 [11] | Physical activity of older adults | Frequency and duration of activities |
| Stanford Brief Physical Activity Survey | Taylor-Piliae et al. 2006 [12] | Physical activity questionnaire | Physical activity on-the-job and during leisure-time during the past year, |
| Canadian Occupational Performance Measure (COPM) | Law et al. 1990 [13] | Individualized outcome measure designed to detect change in a client's self-perception of occupational performance over time. | NA |
| Model of Human Occupation Screening Tool (MOHOST) | Parkinson et al. 2004 [15] | Occupational functioning | Assesses the volition, habituation, skills, and environment |
| London Health and Fitness Questionnaire | Rowland et al. 1994 [14] | Physical activity in older adults | Measured peoples exercise knowledge using a series of positive and negative statements on a 5 point likert scale |
| BRFSS Arthritis Module (PA question) | Remington et al. 1988 [5] | Arthritis related questions | 6 questions measured on a 4 point likert scale |
| MAARS model - motivation to adopt and maintain regular physical activity | NA | NA | NA |
| Adherence to Exercise Scale for Older Patients (AESOP) | Hardage et al. 2007 [16] | Self-efficacy expectations, outcome expectations, and outcome expectancies for predicting adherence | 42 items measuring  1. Self-efficacy expectations (15 items);  2. Outcome expectations (16 items);  3. Outcome expectancies (11 items) |
| Freiburg Questionnaire of Physical Activity | Frey et al. 1998 [17] | NA | NA |
| PRISCUS Physical Activity Questionnaire | Trampisch et al. 2010 [18] | NA | NA |
| Exercise Self-Efficacy | McAuley 1993 [19] | Beliefs in the ability to continue exercise | 8 questions that assess beliefs in one’s ability to continue exercising on a three time per week basis at moderate intensities (upper end of one’s perceived exertion range), for 40+ minutes per session in the future. |
| Confidence in ability to adhere (adaptation of Lorig’s self-efficacy scale | NA | NA | NA |
| UCLA Activity Score | Zahiri et al.1998 [20] | Current activity level | Check one box out of 10 that best describes activity level |
| Home Exercise Compliance assessment (HECA) | NA | Adherence to home exercise | Patients record the number of exercise sessions completed during the previous week. |
| International Physical Activity Questionnaires (IPAQ) short form | Craig et al. 2003 [21] | Physical activity in young and middle aged adults | 4 generic items tested |
| Physical Activity Recall Items | Sallis et al. 1985 [22] | Physical activity | Sleep (2 items) and physical activities (7 items) assessed for the past 7 days |
| Attitudes towards ACL rehabilitation questionnaire | Niven et al 2012 [23] | Attitudes and adherence behaviours to a recommended ACL rehabilitation programme | Assesses intention, attitude, subjective norm, perceived behavioral control, self-efficacy and adherence |
| Health Professional Compliance Evaluation | NA | NA | NA |
| Habitual Physical Activity Questionnaire | Baecke et al. 1982 [24] | Physical activity | 29 items concerning the following five components: occupation, movement, sport, leisure time activities excluding sport, and sleeping habits. |
| Correctness of Exercise Performance Scale | NA | NA | NA |
| Planning for Exercise Scale | Pender 1996 [25] | Commitment to a plan of physical activity | 11 items measuring commitment and strategies to carry out exercise |
| Stages of Exercise Change questionnaire | Reed et al. 1997 [26] | Intention to change or maintain exercise behaviour | Consists of five items representing one of five primary stages of the Trans-theoretical Model |
| Hopkins Rehabilitation Engagement Rating Scale | Kortte et al. 2007 [27] | Used in rating behavioural observation during acute in-patient rehabilitation | 5 items measuring  1.Attendance at rehabilitation session (1); 2.Frequency of required Verbal/Physical Prompts (1); 3.Perceived Positive attitude to exercise (2) ;4.Active participation in rehabilitative exercise (1) |
| The Physical Activity Scale for Individuals with Physical Disabilities | Washburn et al. 2002 [28] | Physical activity | 13 questions record the number of days per week and hours per day for participation in leisure time, household, and occupational physical activities over the past 7 days |
| Physical Activity Level Index | NA | NA | the summed energy expenditure of all reported activities divided by 168, the number of hours per week |
| Arthritis self-management behaviour scale | Lorig et al 1985 [29] | Evidence suggesting that a chronic disease self-management program | NA |
| Aerobics Centre Longitudinal Study Physical Activity Questionnaire (ACLS) | Stofan et al 1998 [30] | Leisure and physical activities | 10 questions which assess participation in 10 specific exercise related activities within the last 3 months |
| Godin Leisure Time Exercise Questionnaire (GLTEQ) | Godin and Sheperd1997 [31] | Leisure time exercise | 2 questions |
| Longitudinal Ageing Study Amsterdam Physical Activity Questionnaire (LAPAQ) | Voorips etal. 1991 [32] | Physical activity | Examines the frequency and duration of specific types of activity in the past two weeks |
| Pittsburgh Rehabilitation Participation Scale | Lenze et al. 2004 [33] | Observed patient participation in a therapy session | 2 items measuring  1.Perceived Intensity/Effort/Exertion (1)  2. Perceived Self-motivation (1) |
| Stanford Exercise Behavior Scale | Lorig et al 1996 [34] | Exercise behaviour | 6 items which assess amount of exercise activities undertaken during the past week |
| Health promoting lifestyle profile | Walket et al. 1987 [35] | Health related questionnaire | 48 item measures health promoting behaviours in 6 domains: nutrition, exercise, health responsibility, stress management, interpersonal support, and self-actualization |
| Health promoting lifestyle II | Walker & Hill-Polerecky 1996 [36] | Health related questionnaire | 52 items in a total scale and six subscales to measure behaviors in the theorized dimensions of health-promoting lifestyle: spiritual growth, interpersonal relations, nutrition, physical activity, health responsibility, and stress management |
| Compliance Behaviour Index | NA | NA | NA |
| Modified - Rehabilitation Adherence Questionnaire (RAQ-M) | Shin et al 1988 [37] | Rehabilitation adherence in injured athletes | 40 items measuring Self-report inventory with subscales designed to assess 1) perceived exertion, 2) pain tolerance, 3) self-motivation, 4) support from significant others, 5) scheduling and 6) environmental conditions. |
| Rehabilitation Compliance Scale (RCS) | Rheiner 1994 [38] | Rehabilitation compliance | Measures patients' compliance to a rehabilitation program. |
| Rehabilitation Over-adherence Questionnaire (ROAQ) | Podlog et al 2013 [39] | Tendency of injured athletes’ toward over- adherence behaviors and their beliefs | Two subscales which measure “ignore practitioner recommendations” (5 items) and “attempt an expedited rehabilitation” (5 items). |

Notes: NA: not available

**Supplementary Table S2: Evidence of reliability for measures of exercise adherence following completion by patients with MSK problems**

| Measure | Evaluations (n) | Internal consistency reliability | COSMIN | Test-retest reliability  (inter-rater; intra-rater; test-retest) | COSMIN |
| --- | --- | --- | --- | --- | --- |
| *Clinician-completed* | | | | | |
| HRERS | 1 | Cronbach’s alpha 0.91  (Kortte et al. 2007)[27] | Poor | *Inter-rater agreement*  2 raters: 1 PT and 1 OT. n=206 patients. Assessment taken at a similar time prior to discharge(specifics not reported)  ICC=0.73  (Kortte et al. 2007)[27] | Poor |
| PRPS | 1 |  |  | *Inter-rater agreement*  Total of 5 therapists (3 PT and 2 OT): 2 therapists independently assessed each session: 20 OT sessions and 25 PT sessions. Therapist pairs were masked to each-others scores.  ICC for OT and PT ratings:  OT: 0.91  PT: 0.96  (Lenze et al. 2004)[33] | Fair |
| SIRAS | 8 | Cronbach’s alpha 0.82 (n= 145)  (Brewer et al. 2000 – Study 1)[6]  Cronbach’s alpha 0.86 for multiple administrations (n= 43)  (Brewer et al. 2000 – Study 1)67] | Fair  Poor | *Intra-rater agreement/ Test-retest:*  2 raters: 1 treating PT and 1 observing PT. n=28 patients.  Re-test period 1 week (stability not reported).  Test-retest reliability (weighted kappa):  Treating physiotherapist: 0.76 ( 95% CI 0.61 to 0.90)  Observing physiotherapist: 0.63 (95% CI 0.39 to 0.88)  (Kolt et al 2007 - study 2)[40]  Number of raters is unclear; n= 31 patients.  Re-test period 1-week (stability unclear).  ICC[2,1]=0.77  (Brewer et al. 2000- study 2)[6]  *Inter-rater agreement:*  2 qualified rehabilitation practitioners; n=12 patients. SIRAS completed after four consecutive appointments (Unclear: re-test period, stability).  Rater Agreement Indices (RAI): 0.94  (Brewer et al. 2002- study 2)[41]  43 student rehabilitation practitioners completed the SIRAS to rate three vignettes of exercise adherence (highly / moderately / minimally adherent)(stability = set vignettes):  Rater Agreement Indices (RAI):  High Adherence 0.90  Moderate adherence 0.86  Low adherence 0.84  Aggregate 0.84  (Brewer et al, 2002 – Study 1)[41]  19 raters; n=43 patients. Inter-rater reliability between primary (n=43 assessments) and secondary provider (n= 39 assessments):  ICC=0.57  (Brewer et al 2000 - study 3)[6]  Three video illustrations of exercise adherence categorized as: High adherence; Moderate adherence; Low adherence:  Inter-rater agreement (Rater Agreement Indices) n= 60 raters:  0.93; 0.87; 0.92  (Kolt et al. 2007- study 1)[40]  2 raters: 1 treating PT and 1 observing PT. n= 28 patients.  Re-test period 1 week.  Inter-rater agreement (weighted kappa) assessed at:  first clinical session 0.76 (95% CI 0.61 to 0.90)  second clinical session 0.89 (95% CI 0.82 to 0.97)  (Kolt et al. 2007- study 2)[40] | Poor  Fair  Poor  Fair  Fair  Poor  Poor |
| *Patient-completed* | | | | | |
| AESOP | 1 |  |  | *Test-retest (interview completion)*  n=28 patients. Re-test period 2-weeks (stability not reported).  AESOP domains ICC (3,1):  Self-efficacy expectations 0.796  Outcome expectations 0.771  Outcome expectancies 0.328  (Hardage et al. 2007)[16] | Poor |
| CHAMPS | 1 |  |  | *Test-retest (self-completion)*  n=173 patients. Re-test period 6 months; (stability expected - non-intervention or control group)  ICC (2,1):  Moderate and greater intensity  Caloric expenditure 0.67  Frequency per week 0.58  All activities  Caloric expenditure 0.66  Frequency per week 0.62  (Stewart et al. 2001)[4] | Poor |
| RAQ-M | 1 | Cronbach’s alpha (n=120):  range –  Perceived exertion 0.66  Pain tolerance 0.79  Environmental conditions 0.79  Support from significant others 0.82  Self-motivation 0.83  Scheduling 0.87  (Shin et al, 2010)[37] | Poor | *Test-retest (self-completion)*  n=120 injured athletes. Re-test 2-weeks; (stability not reported)  ICC: range –  Perceived exertion 0.67  Pain tolerance 0.64  Environmental conditions 0.82  Support from significant others 0.81  Self-motivation 0.78  Scheduling 0.72  (Shin et al, 2010)[37] | Poor |
| ROAQ | 2 | Cronbach's alpha (n=118):  Ignoring practitioner recommendations 0.83  Attempting an expedited rehabilitation 0.70  (Podlog et al 2013 – study 1)[39]  Cronbach's alpha (n=105):  Ignoring practitioner recommendations 0.86  Attempting an expedited rehabilitation 0.75  (Podlog et al 2013 – study 2)[39] | Fair |  |  |

(n): number of studies evaluating the measurement and practical properties of each measure; PT: Physical Therapist; OT: Occupational Therapist; ICC: Intraclass Correlation Coefficient; CI: Confidence Interval; BMI: Body Mass Index; LBF: Lower Body Functioning; 6MW: 6 Minute Walk; SRPF: Self-Reported Physical Functioning; SREF: Self-Reported Energy/Fatigue; SRP: Self-Reported Pain; SRPWB: Self-Reported Psychological Well-Being; HRERS: Hopkins Rehabilitation Engagement Rating Scale; PRPS: Pittsburgh Rehabilitation Participation Scale; SIRAS: Sport Injury Rehabilitation Adherence Scale; AESOP: Adherence to Exercise Scale for Older Patients; CHAMPS: Community Healthy Activities Model Program for Seniors; RAQ-M: Modified - Rehabilitation Adherence Questionnaire; PAF: Principal Axis Factoring; CFA: Confirmatory Factor Analysis; RAOQ: Rehabilitation Over-adherence Questionnaire,

**Supplementary Table S3: Evidence of validity for measures of exercise adherence following completion by patients with MSK problems**

| Measure | Number of evaluations (n) | Known Groups Validity (hypothesis – stated / deduced /not reported?)^a^ | COSMIN | Construct Validity  (structural; construct - divergent / convergent;  hypothesis – stated / deduced /not reported?)^a^ | COSMIN |
| --- | --- | --- | --- | --- | --- |
| *Clinician-completed* | | | | | |
| HRERS | 1 | Groups defined by:  Racial differences (White or Other): N/S  Gender (male or female): N/S  Diagnostic groups (spinal cord injury (SCI), Stroke, Amputation, Orthopaedic): N/S  (Kortte et al. 2007)[27]  Relationship between HRERS (three categories) and clinical variables hypothesized to be associated with engagement (but direction not stated)  Functional Impact Measure (FIM) efficiency  Number of total absences  Number of refusals  Number of non-refusal absences  HRERS (mean) FIM efficiency  <20 / 20-25 / >25 1.25/ 1.87 / 2.03 (p= 0 .04)  HRERS (mean) Total absence rate  <20 / 20-25 / >25 28 / 15 / 9 (p <0.001)  HRERS (mean) Therapy refusal rate  <20 / 20-25 / >25 14 / 7 / 2 (p <0.001)  HRERS (mean) Therapy non-refusal absence rate  <20 / 20-25 / >25 14 / 9 / 7 (p<0.02)  (Kortte et al. 2007) [27] | Fair | *Structural validity*  Factor structure: hypothesized uni-dimensional structure (engagement) supported by principal component factor analysis (explored for each diagnostic group) (n=206).  HRERS with clinical variables (hypothesized association between variables not stated but supported)  Functional Impact Measure (FIM): r= 0.20  Brief Symptom Inventory (depression): r= 0.24  Levine's Denial of Illness questionnaire: r= 0.30  Positive and Affective Negative State( PANAS):  PANAS self-rated negative effect r= 0.23  PANAS self-rated positive effect r= 0.36  Craig Handicap Assessment and Reporting Technique (CHART) - level of functioning at 3 months post discharge: r= 0.22    HRERS with :  Age r= 0.11  Education r=0.16  Length of stay r= 0.13  (Kortte et al. 2007)[27] | Fair  Fair |
| PRPS | 1 |  |  | PRPS with (hypothesized association not stated):  korFunctional Independence Measure-Motor (FIM-motor)  At treatment admission r= 0.38  At treatment discharge: PRPS with change in FIM-motor r= 0.32 (authors suggest the result supports hypothesized association – but this is not explicit)  Gender r=-0.05  Length of stay (LOS) r= -0.13  Age r=-0.21  Race r=-0.01  Medical co-morbidity (count) r= -0.03  (Lenze et al. 2004)[33] | Poor |
| SIRAS | 7 | Three video evaluations describing three levels of adherence: High adherence; Moderate adherence; Low adherence. n=60 assessments.  (hypothesized associations not reported, but can be assumed):  Mean (SD) scores higher for High adherence (mean 13.53 (1.51)); versus Moderate adherence (mean 8.02 (1.95); versus Low adherence (mean 4.59 (1.57)). Statistical significance of group differences not reported.  (Kolt et al, 2007) [40]    Vignettes describing three levels of adherence:  High adherence; Moderate adherence; Low adherence.  (hypothesized associations not reported, but can be assumed):  Statistically significant higher scores (mean (SD)) for High adherence (14.00 (1.27) versus Moderate adherence (8.93 (1.67))(p< 0.001);  and High adherence versus Low adherence (4.79 (1.93)) (p< 0.001).  Statistically significant higher scores for Moderate adherence versus Low adherence (p< 0.001)^b^  (Brewer et al 2002 – study 1)[41] | Poor  Poor | *Structural validity*  Principal component analysis (PCA) supported single factor structure following completion by physiotherapy students (n=60): each student completed the SIRAS for a hypothetical patient across high, medium and low adherence conditions.  (hypothesized structure not proposed)  (Kolt et al, 2007 – study 1)[40]  PCA carried out for two assessors at two sessions supported the single factor structure.  (Kolt et al, 2007 - study 2)[40]  PCA supported the hypothesized single factor structure (single factor 74% of variation) (eigenvalue 2.21) (n= 145)  (Brewer et al, 2000 – study 1)[6]  *Construct validity*  SIRAS scores with attendance at rehabilitation sessions r= 0.21  (association explored but not hypothesized a priori)  (Brewer et al 2000 - study 1)[6] | Poor  Poor  Fair  Poor |
| *Patient-completed* | | | | | |
| AESOP | 1 | Patients with low scores on self-efficacy (n=24) and outcome expectations (n=16) domains adhered to the exercise regime; those with high scores (greater than the mean value) (n=8) did not adhere. However, the external marker for adherence is not clarified.  (Hardage et al, 2007)[16] | Poor | AESOP with SF-12 (version 2) (spearman correlations)  (hypothesized association not stated):  SF-12 Physical Component Score (PCS):  Self-efficacy r=0.13  Outcome expectations r=-0.01  Outcome expectancies r=-0.04  SF-12 Mental Component Score (MCS):  Self-efficacy r=0.01  Outcome expectations r=-0.06  Outcome expectancies r=-0.09  (Hardage et al. 2007)[16] | Poor |
| CHAMPS | 1 | Three groups defined by known activity levels (via a detailed exploration of self-reported activities) (n=249):  Not participating in any exercise or recreational sports. (inactive/initially sedentary)  Participating in some exercise or recreational sports (according to ACSM criteria) (underactive).  Participating in activities at levels that met ACSM guidelines (active)  Hypothesis: levels of physical activity on the CHAMPS would be lowest for the least active.  As hypothesised, the inactive group had statistically significant lower CHAMPS scores (all four values) when compared to the underactive and active groups (p< 0.001).  (Stewart et al. 2001)[4] | Poor | CHAMPS with several health measures (stated hypotheses and supported) (n=249). CHAMPS scores (code):  A. Moderate and greater intensity  A1. Caloric expenditure  A2. Frequency per week  B. All activities  B1. Caloric expenditure  B2. Frequency per week  BMI: range -0.04 (B1) to -0.06 (A1)  Short Physical Performance Battery (lower body functioning): range 0.15 (B2)to 0.28 (A1)  6-minute walk: range 0.10 (B2) to 0.27 (A1)  Short-Form 36-item Health Survey (SF-36) - four domains:  Physical function: range 0.23 (B2) to 0.30 (A1,A2)  Vitality: range 0.10 (B2) to 0.23 (A2)  Body pain: range 0.08 (B2) to 0.17 (A2)  Emotional well-being (range 0.02 (B2) to 0.14 (A2)  (Stewart et al. 2001)[4] | Poor |
| RAQ-M | 1 | Two groups defined by physician opinion: Quick physical recovery (n=20); versus Late physical recovery (n=20): mean (SD) values presented (statistical significance between groups not reported): Group differences:  Self-motivation (0.32)  Scheduling (0.29)  Perceived exertion (0.28)  Support from significant others (0.25)  Pain tolerance (0.19)  Environmental conditions (0.05)  (Shin et al, 2010)[37] | Poor | *Structural validity*  Factor structure: six-domain structure informed by exploratory (n= 102) and then confirmatory (n=120) factor analysis. Hypothesised structure not proposed/defined.  RAQ-M with measures of adherence (hypothesized association not stated): range 0.27 to -0.63.  Patient attendance at rehab sessions – specific result not reported.  SIRAS (3 items):  degree to which a patient exerts themselves: range 0.09 (environmental conditions) to 0.58 (scheduling).  follows practitioner’s instructions and advice: range 0.08 (environmental conditions) to 0.63 (scheduling).  receptive to changes in the rehabilitation program: range 0.14 (environmental conditions) to 0.61 (scheduling)  Self-rated adherence to Home exercise program (HEP) – specific result not reported.  (Shin et al, 2010)[37] | Poor  Poor |
| ROAQ | 2 |  |  | *Structural validity*  Hypothesized a priori 2 factor structure:  Ignoring practitioner recommendations and  Attempting an expedited rehabilitation  Study 1: Following completion of the long-form 19-item measure by injured adolescent athletes (n=118): Principal Axis Factoring (PAF) supported a 2-factor, 10-item solution (2 factors with eigenvalues >1.0; explaining 53.17% of variance) (Podlog et al, 2013 – Study 1)[49]  Study 2: Following completion of the 10-item measure by injured collegiate athletes (n= 105): Confirmatory Factor Analysis (CFA) supported the 2-factor structure (Podlog et al, 2013)[39]  Correlation between the two domains r= 0.49 (Study 1 n= 118) and r= 0.58 (Study 2 n= 105).  *Construct Validity*  *Study 1 (n= 118)(Podlog et al, 2013)[39]*  ROAQ domains with clinical variables (hypothesized association between variables not stated but supported)  Domain 1: Ignoring Practitioner Recommendation with:  Athletic Identity (AIMS): r= 0.23  Appearing Athletically Untalented (SPSQ subscale): r=0.29  Concerns about physical appearance (SPSQ subscale): r= 0.20  Appearing fatigued (SPSQ subscale): r=0.28  Subscale 2: Attempting an Expedited Return with:  Athletic Identity (AIMS): r= 0.46  Appearing Athletically Untalented (SPSQ subscale): r=0.18  *Study 2 (n= 105) (Podlog et al, 2013)[39]*  Subscale 1: Ignoring Practitioner Recommendation with:  Athletic Identity (AIMS): r= 0.27  Appearing fatigued (SPSQ subscale): r=0.22  Mental composure inadequacies(SPSQ subscale): r=0.31  Concerns about Physical Appearance (SPSQ subscale): r=0.36  Subscale 2: Attempting an Expedited Return with:  Concerns about Physical Appearance (SPSQ subscale): r=0.26 | Fair  Fair  Fair  Fair |

^a^Hypothesis testing: hyp deduced - hypothesis of association between variables can be deduced; No hyp - hypothesis of association between variables not stated and cannot be deduced from the article text; ^b^At the end of patients’ first session, the treating Physiotherapist (PT) completed the Sport Injury Rehabilitation Adherence Scale (SIRAS: Brewer et al., 2002) prediction form (n = 169) while SIRAS assessment form was completed at the 6th (n = 100), 12th and last rehabilitation sessions where applicable (n = 23). N: number of studies evaluating the measurement and practical properties of each measure; r: correlation coefficient; LBF: Lower Body Functioning; 6MW: 6 Minute Walk; SF-12: Short-Form 12-item Health Survey; SRPF: Self-Reported Physical Functioning; SREF: Self-Reported Energy/Fatigue; SRP: Self-Reported Pain; SRPWB: Self-Reported Psychological Well-Being; HRERS: Hopkins Rehabilitation Engagement Rating Scale; PRPS: Pittsburgh Rehabilitation Participation Scale; SIRAS: Sport Injury Rehabilitation Adherence Scale; AESOP: Adherence to Exercise Scale for Older Patients; CHAMPS: Community Healthy Activities Model Program for Seniors; RAQ-M: Modified - Rehabilitation Adherence Questionnaire; ACSM: American College of Sports Medicine; PAF: Principal Axis Factoring; CFA: Confirmatory Factor Analysis; SPSQ: Self-Presentation in Sport Questionnaire; AIMS: Athletic Identity Measurement Scale; ROAQ: Rehabilitation Over-adherence Questionnaire; I-PRRS: Modified Injury Psychological Readiness to Return to Sport Scale.

**References:**

1. Terwee CB, Jansma EP, Riphagen II, de Vet HC. Development of a methodological PubMed search filter for finding studies on measurement properties of measurement instruments. Quality of Life Research 2009;18:1115-1123.]
2. Topolski TD, LoGerfo J, Patrick DL, Williams B, Walwick J, Patrick MM. Peer reviewed: the Rapid Assessment of Physical Activity (RAPA) among older adults. Preventing chronic disease. 2006 Oct;3(4).
3. Dannecker EA, Hausenblas HA, Connaughton DP, Lovins TR. Validation of a stages of exercise change questionnaire. Research Quarterly for Exercise and Sport. 2003 Sep 1;74(3):236-47.
4. Stewart AL. Community Health Activities Model Program for Seniors. Institute for Health and Aging, University of California San Francisco. 1998.
5. Remington PL, Smith MY, Williamson DF, Anda RF, Gentry EM, Hogelin GC. Design, characteristics, and usefulness of state-based behavioral risk factor surveillance: 1981-87. Public health reports. 1988 Jul;103(4):366.
6. Brewer BW, Van Raalte JL, Petitpas AJ, Sklar JH, Pohlman MH, Krushell RJ, Ditmar TD, Daly JM, Weinstock J. Preliminary psychometric evaluation of a measure of adherence to clinic-based sport injury rehabilitation. Physical Therapy in Sport. 2000 Aug 31;1(3):68-74.
7. Wendel-Vos GC, Schuit AJ. Short Questionnaire to ASSess Health enhancing physical activity. Bilthoven, The Netherlands: National Institute of Public Health and the Environment. Center for Chronic Diseases Epidemiology. 2002.
8. Tegner Y, Lysholm J. Rating systems in the evaluation of knee ligament injuries. Clinical orthopaedics and related research. 1985 Sep 1;198:42-9.
9. Washburn RA, Smith KW, Jette AM, Janney CA. The Physical Activity Scale for the Elderly (PASE): development and evaluation. Journal of clinical epidemiology. 1993 Feb 1;46(2):153-62.
10. Pereira MA, FitzerGerald SJ, Gregg EW. A collection of physical activity questionnaires for health-related research. Kriska and Caspersen, Eds. Centers for Disease Control and Prevention. Med Sci Sports Exerc. 1997;29(6 Suppl):S1-205.
11. Dipietro L, Caspersen CJ, Ostfeld AM, Nadel ER. A survey for assessing physical activity among older adults. Medicine & Science in Sports & Exercise. 1993 May.
12. Taylor-Piliae RE, Norton LC, Haskell WL, Mahbouda MH, Fair JM, Iribarren C, Hlatky MA, Go AS, Fortmann SP. Validation of a new brief physical activity survey among men and women aged 60–69 years. American journal of epidemiology. 2006 Sep 15;164(6):598-606.
13. Law M, Baptiste S, McColl M, Opzoomer A, Polatajko H, Pollock N. The Canadian occupational performance measure: an outcome measure for occupational therapy. Canadian Journal of Occupational Therapy. 1990 Apr 1;57(2):82-7.
14. Parkinson S, Forsyth K, Kielhofner G. A user's manual for the Model of Human Occupation Screening Tool (MOHOST). Model of Human Occupation Clearinghouse, Department of Occupational Therapy, College of Applied Health Sciences, University of Illinois at Chicago; 2004.
15. Rowland L, Dickinson EJ, Newman P, Ford D, Ebrahim S. Look After Your Heart programme: impact on health status, exercise knowledge, attitudes, and behaviour of retired women in England. Journal of Epidemiology and Community Health. 1994 Apr 1;48(2):123-8.
16. Hardage J, Peel C, Morris D, Graham C, Brown CJ, Foushee RH, Braswell J. Adherence to exercise scale for older patients (AESOP): a measure for predicting exercise adherence in older adults after discharge from home health physical therapy. Journal of Geriatric Physical Therapy. 2007 Aug 1;30(2):69-78.
17. Frey I, Berg A, Grathwohl D, Keul J. [Freiburg Questionnaire of physical activity--development, evaluation and application]. Sozial-und Praventivmedizin. 1998 Dec;44(2):55-64.
18. Trampisch U, Platen P, Burghaus I, Moschny A, Wilm S, Thiem U, Hinrichs T. [Reliability of the PRISCUS-PAQ. Questionnaire to assess physical activity of persons aged 70 years and older]. Zeitschrift fur Gerontologie und Geriatrie. 2010 Dec;43(6):399-406.
19. McAuley E. Self-efficacy and the maintenance of exercise participation in older adults. Journal of behavioral medicine. 1993 Feb 1;16(1):103-13.
20. Zahiri CA, Schmalzried TP, Szuszczewicz ES, Amstutz HC. Assessing activity in joint replacement patients. The Journal of arthroplasty. 1998 Dec 31;13(8):890-5.
21. Craig CL, Marshall AL, Sjöström M, Bauman AE, Booth ML, Ainsworth BE, Pratt M, Ekelund U, Yngve A, Sallis JF, Oja P. and the IPAQ Consensus Group and the IPAQ Reliability and Validity Study Group. International Physical Activity Questionnaire (IPAQ): 12-country reliability and validity. Med Sci Sports Exerc. 2003;35(13):81-95.
22. Sallis JF, Haskell WL, Wood PD, Fortmann SP, Rogers T, Blair SN, Paffenbarger RS. Physical activity assessment methodology in the Five-City Project. American journal of epidemiology. 1985 Jan 1;121(1):91-106.
23. Niven A, Nevill A, Sayers F, Cullen M. Predictors of rehabilitation intention and behavior following anterior cruciate ligament surgery: an application of the Theory of Planned Behavior. Scandinavian journal of medicine & science in sports. 2012 Jun 1;22(3):316-22.
24. Baecke JA, Burema J, Frijters JE. A short questionnaire for the measurement of habitual physical activity in epidemiological studies. The American journal of clinical nutrition. 1982 Nov 1;36(5):936-42.
25. Pender, N.J., 1996. Health Promotion in Nursing Practice, third ed. Appleton & Lange, Stamford, CT.
26. Reed GR, Velicer WF, Prochaska JO, Rossi JS, Marcus BH. What makes a good staging algorithm: examples from regular exercise. American Journal of Health Promotion. 1997 Sep 1;12(1):57-66.
27. Kortte KB, Falk LD, Castillo RC, Johnson-Greene D, Wegener ST. The Hopkins rehabilitation engagement rating scale: development and psychometric properties. Archives of physical medicine and rehabilitation. 2007 Jul 31;88(7):877-84.
28. Washburn RA, Zhu W, McAuley E, Frogley M, Figoni SF. The physical activity scale for individuals with physical disabilities: development and evaluation. Archives of physical medicine and rehabilitation. 2002 Feb 28;83(2):193-200.
29. Lorig K, Lubeck D, Kraines RG, Seleznick M, Holman HR. Outcomes of self-help education for patients with arthritis. Arthritis and Rheumatism, 1985, 28, 680—685.
30. Stofan JR, DiPietro L, Davis D, Kohl 3rd HW, Blair SN. Physical activity patterns associated with cardiorespiratory fitness and reduced mortality: the Aerobics Center Longitudinal Study. American Journal of Public Health. 1998 Dec;88(12):1807-13.
31. Godin G, Shephard RJ. Godin leisure-time exercise questionnaire. Med Sci Sports Exerc. 1997 Jun;29(6):36-8.
32. Voorrips LE, Ravelli AC, Petra C, Dongelmans A, Deurenberg P, van Staveren WA. A physical activity questionnaire for the elderly. Diet and physical activity as determinants of nutritional status in elderly women. 1991 Aug 1:43.
33. Lenze EJ, Munin MC, Quear T, Dew MA, Rogers JC, Begley AE, Reynolds CF. The Pittsburgh Rehabilitation Participation Scale: reliability and validity of a clinician-rated measure of participation in acute rehabilitation. Archives of physical medicine and rehabilitation. 2004 Mar 31;85(3):380-4.
34. Lorig K, Stewart A, Ritter P, González V, Laurent D, & Lynch J, Outcome Measures for Health Education and other Health Care Interventions. Thousand Oaks CA: Sage Publications, 1996, pp.25,37-38.
35. Walker SN, Sechrist KR, Pender NJ. The health-promoting lifestyle profile: development and psychometric characteristics. Nursing research. 1987 Mar 1;36(2):76-81.
36. Walker SN, Hill-Polerecky DM. Psychometric evaluation of the health-promoting lifestyle profile II. Unpublished manuscript, University of Nebraska Medical Center. 1996 Jun 13:120-26.
37. Shin JT, Park R, Song WI, Kim SH, Kwon SM. The redevelopment and validation of the Rehabilitation Adherence Questionnaire for injured athletes. International Journal of Rehabilitation Research. 2010 Mar 1;33(1):64-71.
38. Rheiner NW. Validity and reliability of the Rehabilitation Compliance Scale. Mind Garden, Palo Alto, CA. 1994.
39. Podlog L, Gao Z, Kenow L, Kleinert J, Granquist M, Newton M, Hannon J. Injury rehabilitation overadherence: preliminary scale validation and relationships with athletic identity and self-presentation concerns. Journal of athletic training 2013; 48(3): 372-381.
40. Kolt GS, Brewer BW, Pizzari T, Schoo AM, Garrett N. The sport injury rehabilitation adherence scale: A reliable scale for use in clinical physiotherapy. Physiotherapy 2007;93:17-22.
41. Brewer BW, Avondoglio JB, Cornelius AE, Van Raalte JL, Brickner JC, Petitpas AJ, Kolt GS, Pizzari T, Schoo AM, Emery K. Construct validity and interrater agreement of the sport injury rehabilitation adherence scale. J.Sport Rehab. 2002;11:170-178.
